# Optimized CEST cardiovascular magnetic resonance for assessment of metabolic activity in the heart

**DOI:** 10.1186/s12968-017-0411-1

**Published:** 2017-11-30

**Authors:** Zhengwei Zhou, Christopher Nguyen, Yuhua Chen, Jaime L. Shaw, Zixin Deng, Yibin Xie, James Dawkins, Eduardo Marbán, Debiao Li

**Affiliations:** 1 Biomedical Imaging Research Institute, Cedars-Sinai Medical Center, 8700 Beverly Blvd. PACT Suite 400, Los Angeles, CA 90048 USA; 2 Department of Bioengineering, University of California Los Angeles, Los Angeles, CA USA; 3 Cardiovascular Research Center, Massachusetts General Hospital, Charlestown, MA USA; 4 Harvard Medical School, Boston, MA USA; 5 Heart Institute, Cedars-Sinai Medical Center, Los Angeles, CA USA

**Keywords:** MRI, Cardiac CEST, Creatine, Metabolic imaging, CEST CMR

## Abstract

**Background:**

Previous studies have linked cardiac dysfunction to loss of metabolites in the creatine kinase system. Chemical exchange saturation transfer (CEST) is a promising metabolic cardiovascular magnetic resonance (CMR) imaging technique and has been applied in the heart for creatine mapping. However, current limitations include: (a) long scan time, (b) residual cardiac and respiratory motion, and (c) B_0_ field variations induced by respiratory motion. An improved CEST CMR technique was developed to address these problems.

**Methods:**

Animals with chronic myocardial infarction (*N* = 15) were scanned using the proposed CEST CMR technique and a late gadolinium enhancement (LGE)  sequence as reference. The major improvements of the CEST CMR technique are: (a) Images were acquired by single-shot FLASH, significantly increasing the scan efficiency. (b) All images were registered to reduce the residual motion. (c) The acquired Z-spectrum was analyzed using 3-pool-model Lorentzian-line fitting to generate CEST signal, reducing the impact of B_0_ field shifting due to respiratory motion. Feasibility of the technique was tested in a porcine model with chronic myocardial infarction. CEST signal was measured in the scar, border zone and remote myocardium. Initial studies were performed in one patient.

**Results:**

In all animals, healthy remote myocardial CEST signal was elevated (0.16 ± 0.02) compared to infarct CEST signal (0.09 ± 0.02, *P* < 0.001) and the border zone (0.12 ± 0.02, *P* < 0.001). For both animal and patient studies, the hypointense regions in the CEST contrast maps closely match the bright areas in the LGE images.

**Conclusions:**

The proposed CEST CMR technique was developed to address long scan times, respiratory and cardiac motion, and B_0_ field variations. Lower CEST signal in bright region of the LGE image is consistent with the fact that myocardial infarction has reduced metabolic activity.

## Background

Adenosine triphosphate (ATP) is an essential energy source that governs myocardial contraction [1]. The creatine kinase (CK) system plays a vital role in the synthesis of myocardial ATP. ATP is generated from the conversion of phosphocreatine (PCr) and adenosine diphosphate (ADP) to creatine (Cr) catalyzed by CK. Previous studies have linked cardiac dysfunction to the loss of metabolites in the CK system [2–4]. The measurement of Cr or PCr can serve as a biomarker to investigate the metabolic change in the myocardium [5–9].

Chemical exchange saturation transfer (CEST) is an emerging MRI technique for metabolic imaging [10]. By saturating the solute protons, water signal will also drop because of the constant exchange between solute protons and water protons [11]. It has been shown that CEST can be used to map Cr distribution because of chemical transfer between its amine protons (−NH_2_) and water protons. CEST can detect Cr separately from other CK metabolites because Cr protons alone have an intermediate transfer rate with water protons [12–14]. Compared to MR spectroscopy techniques, CEST yields better spatial resolution and higher sensitivity because of indirect detection via water protons [14, 15].

CEST has been applied in the heart for in vivo endogenous Cr imaging in animal studies [16–19]. Haris et al. was the first to show the feasibility of high-spatial-resolution mapping of Cr using CEST cardiovascular magnetic resonance (CMR). However, the method poses some technical challenges which could potentially hinder the clinical translation of the CEST CMR technique: (a) long scan time (~50 min per slice), (b) residual cardiac and respiratory motion, and (c) B_0_ field variations mostly induced by respiratory motion.

In this study, we developed a clinically translatable CEST CMR technique with significantly reduced scan time (~5 min), improved motion registration and CEST signal analysis to address the aforementioned challenges. The proposed technique was then validated in chronic myocardial infarction porcine model using late gadolinium enhancement (LGE) as a reference. This animal model was chosen because it has been clearly shown that scar regions have reduced metabolic activity level compared to remote myocardium [20]. A repeatability study was performed in healthy subjects, and an initial patient study is presented.

## Methods

### Animal study

All animal-related procedures were approved by the Institutional Animal Care and Use Committee (IACUC) at Cedars-Sinai Medical Center. Myocardial infarction was induced in 15 Yucatan minipigs following the procedure described in a previous study [21]. Specifically, animals were sedated by intramuscular ketamine 20 mg/kg, acepromazine 0.25 mg/kg and atropine 0.05 mg/kg, followed by 10 mL intravenous thiopental. Endotracheal intubation was then performed, and anesthesia maintained by ventilation with 1% to 2% isoflurane. Catheters were inserted through the left carotid artery. Coronary X-ray angiography was performed to visualize the coronary arteries and identify the site for occlusion. An anteroseptal myocardial infarction was induced by inflation of an angioplasty balloon in the mid-left anterior descending artery to cause coronary occlusion for 2.5 h. Finally, the animals were taken to the post-op recovery room. All imaging was performed eight weeks after the infarction.

Animal study (*N* = 15) was performed on a 3 Tesla clinical scanner (Magnetom Verio; Siemens Healthineers, Erlangen, Germany) using a 12-channel phase array coil for data acquisition. Throughout the imaging procedures, anesthesia was maintained with isoflurane (1–3.5%).

The imaging protocol included balanced steady state free precession (bSSFP) cine, CEST CMR and LGE. bSSFP cine was performed to localize the quiescent period of the cardiac cycle (1.4 × 1.4 × 6 mm^3^; electrocardiogram-gated; 35 cardiac phases; TR/TE = 3.2/1.6 ms; flip angle 50°). LGE imaging was completed 15 min after the contrast agent injection (0.1 mmol/kg, gadobutrol, Gadovist, Bayer Healthcare, Berline, German) using phase sensitive inversion recovery FLASH (1.3 × 1.3 × 6 mm^3^; TR/TE/TI = 362/1.5/335 ms; flip angle 20°). All imaging was performed in the short axis plane at three mid-ventricular slice locations of the left ventricle. Breath hold was controlled by a ventilator in cine imaging and LGE imaging.

**Fig. 1:**
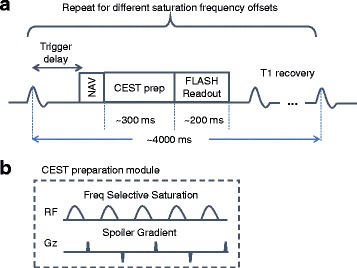
(**a**) Pulse sequence diamgram of the optimized CEST CMR techniue. (**b**) CEST preparation module used in the proposed CEST CMR sequence

CEST CMR scans were performed before the contrast injection using FLASH readout (resolution 2.1 × 2.1 mm^2^; slice thickness 6–8 mm; TR/TE = 4000/1.5 ms; flip angle 12°). Figure 1 shows the pulse sequence diagram of the proposed CEST CMR technique. Electrocardiogram triggering and navigator gating were used to reduce the effects of cardiac and respiratory motion. Each image was acquired by single-shot FLASH (~200 ms readout block placed in quiescent period). Thirty-three images were collected at different saturation frequency offsets ranging from −4.8 ppm to 4.8 ppm with a step size of 0.3 ppm. The CEST preparation module consists of five Gaussian-shaped pulses with 2700° flip angle and 30 ms duration at a duty cycle of 50% (the equivalent B_1_ power is 3.76 uT). The spoiler gradient was altered in different directions to crush the residual transverse magnetization. An additional image was acquired without CEST saturation for normalization reference. The scan time for each slice was approximately 5 min with 40% navigator efficiency.

CEST CMR technique was optimized in the following aspects: (a) Images were acquired by single-shot FLASH instead of segmented acquisition, significantly increasing the scan efficiency. (b) All images were registered to reduce the residual left ventricle wall motion (up to 4 mm myocardial displacement) using Medical Imaging Registration Toolbox (MIRT) [22]. (c) The acquired Z-spectrum was fitted to the Lorentzian-shaped 3-pool-model to increase the reliability of the generated CEST contrast map by reducing the impact of signal fluctuation from B_0_ field shifting introduced by respiratory motion.

### Healthy subject study

CEST CMR in healthy subjects (*n* = 14) was performed on a 3 T clinical scanner (Magnetom Verio; Siemens Healthineers) using a 32-channel phase array coil for data acquisition. For the healthy subject and patient imaging studies, the protocol was approved by Institutional Review Board at Cedars-Sinai Medical Center and written informed consent was obtained from each subject before the exam. For each healthy subject, repeatability of the proposed CEST CMR method was evaluated in two mid-ventricular slices by repeating the scan twice in the same imaging position.

The parameters of CEST CMR sequence used in healthy subject study were similar compared to the animal study. Each image was acquired by single-shot FLASH (Field-of-view: 350 × 280 mm^2^; spatial resolution: 2.7 × 2.2 × 8.0 mm^3^; TR/TE = 4000/1.45 ms; matrix size: 160 × 104; iPAT: 2; flip angle: 12°; acquisition window: 163 ms). 3 Gaussian-shaped pulses with flip angle of 2700° and pulse duration of 80 ms were used in the CEST preparation module. The interpulse delay was 80 ms. Equivalent RF irradiation power is 1.90 uT. CEST saturation frequency offsets range from −3.6 ppm to 3.6 ppm with a step size 0.3 ppm. An additional image was acquired without CEST saturation for normalization reference. The scan time for each slice was approximately 5 min with 30% navigator efficiency.

### Image analysis

Post processing was performed with custom-written programs in Matlab (The Mathworks, Natick, Massachusetts, USA). For each pixel, the Z-spectrum was generated as the signal intensity of each image acquired at different saturation frequency offsets normalized by the reference image. By using 3-pool-model Lorentzian-line fitting, the acquired Z-spectrum was separated into CEST, direct water saturation (DWS), and conventional magnetization transfer (MT), three major effects in saturation experiments with aqueous solutions [16, 23]. The center of the DWS curve is the central water frequency, which represents B_0_ field information. The distance between the center of the CEST curve and DWS curve is constantly +1.8 ppm, which is the resonant frequency of amine protons. CEST signal is defined as the amplitude of the fitted CEST curve.

CEST maps were generated using pixel-by-pixel Z-spectrum fitting in the myocardium. In the animal study, regions of interest (ROIs) were placed in the scar region, remote myocardium and border zone adjacent to the infarcted myocardium using LGE as reference. The average CEST signal was determined in scar, border zone and healthy remote myocardium, for each subject. Comparisons were performed using repeated measures one-way ANOVA analysis followed by Tukey’s multiple comparisons test in GraphPad Prism 6 (Graph-Pad Software, La Jolla, California, USA). A two-tailed value of *p* < 0.05 was considered to be statistically significant. In the healthy subject studies, the average CEST signal was calculated in the entire myocardium (“global”) and in the septum. The repeatability was evaluated by performing Bland-Altman plot in GraphPad Prism 6 (GraphPad Software).

### Patient study

One patient who was referred for LGE CMR exam for evaluation of chronic myocardial infarction was studied using the proposed CEST CMR sequence with the same settings/parameters as the volunteer studies. LGE imaging was completed 15 min after the contrast agent injection (0.1 mmol/kg, gadobutrol, Gadovist, Bayer Inc.) using phase sensitive inversion recovery FLASH (1.4 × 1.4 × 8 mm^3^; TR/TE/TI = 330/1.6/300 ms; flip angle 20°).

## Results

### Animal study

In total, the CEST CMR scans were performed in 15 animals. The heart rates of the animals were in the range of 100–120 beats/min during the CMR scan.

**Fig. 2:**
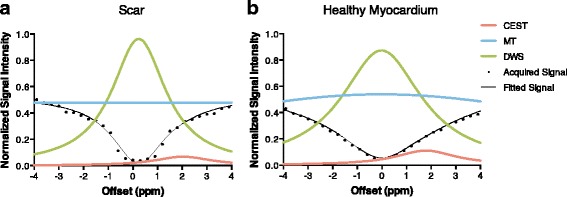
Z-spectrum of (**a**) scar region and (**b**) healthy myocardium. By using the 3-pool-model fitting, Z-spectrum (black) was separated into CEST curve (pink), MT curve (blue) and DWS curve (green). The center of DWS curve represents the resonant water frequency (B_0_ field). The peak of CEST curve was defined as the CEST signal. It is clear that the healthy myocardium has higher CEST signal than scar, which suggests more Cr distribution in the healthy myocardium

To separate the CEST signal from MT and DWS effects, we used 3-pool-model fitting to analyze the Z-spectrum (Fig. 2). The peak of the CEST curve is defined as CEST signal, which is used in the following quantitative analysis. Comparing the Z-spectrum analysis curves in the scar with those in the healthy myocardium, it is clear that CEST signal is lower in the scar region.

**Fig. 3:**
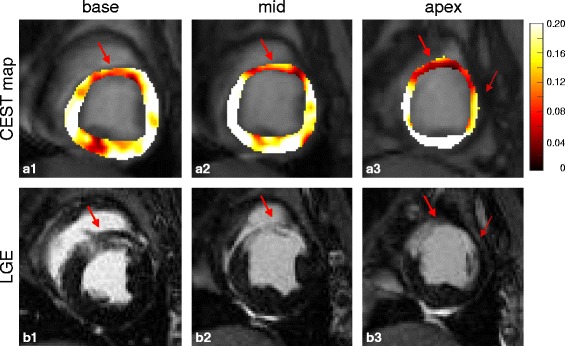
(**a**) CEST maps and (**b**) corresponding LGE images of one representative subject in three slice locations. The hypointense regions (arrows) in the CEST maps match the LGE positive regions (arrows)

Figure 3 shows typical CEST contrast maps and corresponding LGE images in three slice locations. The infarcted regions have lower CEST signal than healthy remote myocardium. Spatially, the hypointense regions in the CEST contrast maps closely match the bright areas in LGE images.

**Fig. 4:**
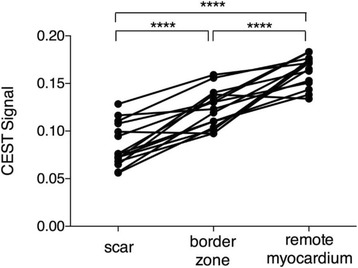
CEST signal in the scar (0.09 ± 0.02) is significantly reduced compared to healthy remote myocardium (0.16 ± 0.02), *p* < 0.001. CEST signal in the border zone (0.12 ± 0.02) was also reduced compared to remote myocardium, *p* < 0.001

In all animals, infarct CEST signal (0.09 ± 0.02) is significantly reduced compared to the CEST signal found in the healthy remote myocardium (0.16 ± 0.02), *p* < 0.001, as shown in Fig. 4. The CEST signal in the border zone (0.12 ± 0.02) was also reduced compared to the remote myocardium, *p* < 0.001, while it was elevated compared to the infarcted tissue, *p* < 0.001.

### Healthy subject study

Fourteen healthy subjects (10 male, 39 ± 13 years, BMI 24 ± 4) were included in the studies. The average scan time of each slice was 4.8 ± 2.8 min.

**Fig. 5:**
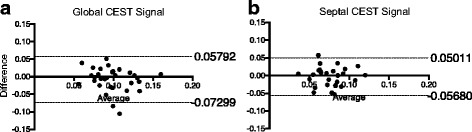
Bland-Altman plots of (**a**) global CEST signal and (**b**) septal CEST signal. The dashed lines represent 95% limits of agreement

The Bland-Altman plots of global CEST signal and septal CEST signal were shown in Fig. 5a and b, respectively. The 95% limits of agreement of the global CEST signal were −0.07 to 0.06, while the 95% limits of agreement of the septal CEST signal were −0.06 to 0.05.

### Patient study

**Fig. 6:**
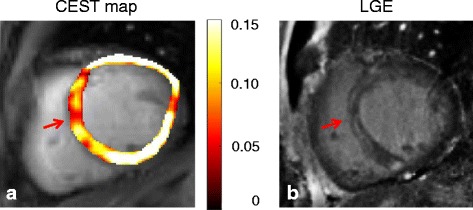
(**a**) CEST map and (**b**) corresponding LGE image in the patient with a chronic myocardial infarction

The CEST map and the corresponding LGE image of the patient study were shown in Fig. 6. The hypointense region in the CEST maps is consistent with the scar region shown on the LGE image.

## Discussion

In this work, we developed a CEST CMR technique with significantly reduced scan time (~5 min), improved motion registration and CEST signal analysis to show the feasibility of the proposed technique in detecting Cr distribution in the myocardium in a clinically feasible scan time. The animal study showed decreased CEST signal in infarcted region compared to remote myocardium, which is consistent with previous finding [16]. The repeatability healthy subject studies and the preliminary patient result also suggest initial feasibility of the proposed CEST CMR technique.

As CK metabolites are essential in providing energy for myocardial contraction, cardiac metabolic impairment is now considered as a cause, rather than a result of cardiac diseases [24]. Detection of CK metabolites such as creatine can potentially help the clinical diagnosis and treatment of cardiovascular diseases. It can be used to further understand the underlying mechanisms of metabolic change processes in these diseases as well as to evaluate therapy efficiency and guide therapy optimization.

In this study, we compared the CEST signal in the scar, border zone and remote myocardium. As expected, the CEST signal in the scar was significantly reduced compared to the CEST signal in the remote myocardium. This is consistent with the previous findings that the infarcted tissue has reduced metabolic activity [20]. More interestingly, the CEST signal in the border zone was also significantly reduced compared to the remote myocardium. This potentially suggests that the border zone might also have reduced myocardial metabolic activity. However, a more comprehensive study needs to be performed to further demonstrate this point.

Cardiac MR Spectroscopy (CMRS) is also capable of detecting the activity of CK metabolites in the myocardium. PCr and ATP can be detected as two peaks in ^31^P spectrum. The ratio of PCr over ATP can serve as an indicator of myocardial energy metabolism [5, 25]. In ^1^H spectrum, Cr and PCr have the same chemical shift and only their combined total amount can be measured [6, 26]. However, these techniques have not gained common application over the years because of limitations such as low spatial resolution, lengthy scan time, and complicated acquisition protocol. ^31^P CMRS also requires additional hardware, which limits its availability. All of these issues hinder the clinical translation of CMRS approaches. However, they can be potentially addressed by CEST CMR techniques. In this study, we have shown the feasibility of CEST CMR technique with typical CMR spatial resolution and clinically acceptable scan time. CEST CMR is also more sensitive compared to CMRS techniques because the signal is indirectly detected from the water pool. Without any special requirement for hardware or technician training, this technique can be easily translated into clinical studies.

The CEST CMR sequence we proposed in this study is significantly faster than the previous technique [16]. One of the major reasons is that we used single-shot FLASH readout instead of segmented readout. In addition to its contribution to speed acceleration, single-shot readout also minimizes the respiratory motion within the acquisition of each CEST-weighted image at different saturation frequency offsets. This reduces the motion artifacts and B_0_ field changes within the acquisition of each single image. It should be noted, though, when the subject has a relatively high heart rate and short quiescent period, the image acquired using single-shot readout will be blurrier. More advanced image reconstruction methods can be used in the future to shorten the acquisition period.

It is known that the heart is one of the most difficult organs for imaging because of constant cardiac motion and respiratory motion. In CEST CMR scans, in addition to the motion within the acquisition of one image that leads to blurriness and the misregistration between different images that causes error in pixel-by-pixel mapping, we are also facing B_0_ field variations caused by residual motion. Respiratory phase mismatch, even within the navigator acceptance window, leads to B_0_ field variations in the myocardium for images acquired with different saturation frequency offsets. Because of the relative position of heart (tissue) and lung (air) in the lateral wall, the B_0_ field is changing rapidly in the heart, especially the lateral wall region. In this study, 3-pool-model fitting is used to generate CEST signal so the B_0_ field variations can be treated as noise. However, this method can address this issue only to certain extent. Therefore, sometimes inhomogeneity is observed in the CEST map (Fig. 3), especially in the lateral wall region. This issue can be potentially addressed using advanced shimming coil design so the B_0_ field over the myocardium can be more homogeneous. Another potential method could be incorporating additional B_0_ field information in the data acquisition.

This study shows the feasibility of CEST CMR technique in healthy subjects and animal studies. Preliminary patient result is also presented. However, to further demonstrate the utility of CEST CMR technique in patients, more comprehensive studies need to be performed. The animal model used in this study only yielded transmural infarcts. Non-transmural infarcts are typically smaller in size and may be more difficult to detect. Whether the spatial resolution and sensitivity of the current technique is enough for non-transmural infarcts requires more studies. Additionally, the data acquired in this study were limited to the left anterior descending territory. Further studies will be needed to evaluate the performance of CEST CMR at different arterial territories.

In this study, we applied CEST CMR in the well-studied chronic infarction model using LGE as reference. However, CEST CMR should not be seen as a potential replacement for LGE for scar quantification. As a supplement, CEST CMR provides the unique metabolic information which could potentially be used to study the undergoing mechanisms in cardiac dysfunctions. However, more comprehensive studies still need to be performed to demonstrate the utility of CEST CMR. The results in this study show that the infarct region has about 50% drop in CEST signal compared to healthy remote myocardium. In some types of cardiac dysfunctions which have mild metabolic impairment, the difference of signal could be smaller. Further investigation is needed to evaluate whether CEST CMR technique has the reproducibility and sensitivity to detect cardiac dysfunctions with mild metabolic abnormalities. Histopathology studies are also needed to demonstrate the CEST signal change is correlated to creatine distribution in the myocardium.

## Conclusion

We developed a clinically feasible CEST CMR technique that is significantly faster than the previous approach. In a chronic myocardial infarction pig model, we demonstrated the proposed CEST CMR technique can discern differences in the CEST signal between infarct and remote myocardium. This study shows the feasibility of CEST CMR technique in healthy subjects, animal studies, and a preliminary patient study.

## Abbreviations

ADP: Adenosine diphosphate
ATP: Adenosine triphosphate
bSSFP: Balanced steady state free precession
CEST: Chemical exchange saturation transfer
CK: Creatine kinase
CMR: Cardiovascular magnetic resonance
Cr: Creatine
DWS: Direct water saturation
FLASH: Fast low angle shot
LGE: Late gadolinium enhancement
MT: Magnetization transfer
PCr: Phosphocreatine
ROI: Region of interest
